# Malignant rhabdoid tumor in the renal allograft of an adult transplant recipient: a unique case of a rare tumor

**DOI:** 10.1186/s13000-017-0677-5

**Published:** 2017-12-19

**Authors:** Jing Xiong, Tiefen Su, Pengcheng Zhu, Qilin Ao, Qiurong Ruan, Guoping Wang

**Affiliations:** 1 Institute of Pathology, Tongji Hospital, Tongji Medical College, Huazhong University of Science and Technology, 1095 Jiefang Avenue, Wuhan, 430030 People’s Republic of China; 2 Department of Pathology, School of Basic Medical Science, Tongji Medical College, Huazhong University of Science and Technology, Wuhan, 430030 People’s Republic of China

**Keywords:** Malignant rhabdoid tumor (MRT), SMARCB1/INI1, Kidney transplantation, Donor-to-recipient malignancy transmission

## Abstract

**Background:**

Renal transplant recipients have increased risk for developing malignant diseases because of immunosuppression or donor-to-recipient transmission. Malignant rhabdoid tumor (MRT) is a rare, highly aggressive and lethal tumor primarily affecting the kidney of infants and young children. MRT has not been reported in the renal allograft of an adult recipient after kidney transplantation.

**Case presentation:**

In this report, a 47-year-old woman who received a kidney transplantation from an infant donor and developed a mass in the transplanted kidney is presented. Pathological examinations revealed a malignant tumor with rhabdoid cells morphologically and the loss of INI1 expression immunohistochemically. The diagnosis of malignant rhabdoid tumor in the transplanted kidney was made. We confirmed that donor-to-recipient malignancy transmission was the cause of MRT in the transplanted kidney by fluorescence in situ hybridization (FISH) and short tandem repeat (STR) analysis.

**Conclusion:**

To our knowledge, this is the first case of MRT in an adult renal allograft recipient. This report highlights the importance of the criteria for selection of donors to screen possible malignant tumors transmission.

## Background

Graft survival has improved considerably in kidney transplantation thanks to potent immunosuppression. On the other hand, the overall incidence of malignancy after renal transplantation is much higher than in the general population [1]. The major reasons for this increased risk are thought to be perturbation of immune surveillance mechanisms secondary to the chronic use of immunosuppressive agents and donor-to-recipient malignancy transmission [2]. Malignant rhabdoid tumor (MRT) of the kidney is a highly aggressive tumor of infancy and childhood [3]. This tumor is characterized by noncohesive tumor cells with eccentric nuclei and eosinophilic cytoplasm morphologically and deletion/mutation of the *SMARCB1/INI1* gene located on chromosome 22q 11.2 genetically [4]. Here, we report a rare case of MRT arising from a renal allograft in a 47-year-old female patient who received a kidney transplantation for renal failure. We confirmed that the donor-to-recipient malignancy transmission was the cause of MRT in the transplanted kidney by fluorescence in situ hybridization (FISH) and short tandem repeat (STR) analysis. To our knowledge, this is the first case of MRT in an adult renal allograft recipient.

Approval was obtained from Ethics committee of Tongji Hospital, Tongji Medical College, Huazhong University of Science and Technology. Written informed consent was obtained from the patient in this study.

## Case presentation

### Case report

A female patient was diagnosed with thrombocytopenia due to persistent gum bleeding and lower extremity congestion at the age of 40 years in a local hospital. Subsequently, she was detected to have hypertension. She took nifedipine for hypertension for three years, but her health conditions worsened. A renal function examination showed an elevated serum creatinine (Scr) level (342 μmol/l), and symptomatic treatment did not improve renal function before dialysis. After six months of dialysis, the patient underwent kidney transplant operation since a 56-day-old infant who died from a central nerve system (CNS) tumor (suspected astrocytoma without pathological evidence) came to the hospital as a kidney donor by his parents and was found to be suitable for kidney donation. There was no history of rhabdoid tumors in the donor family history.

**Fig. 1:**
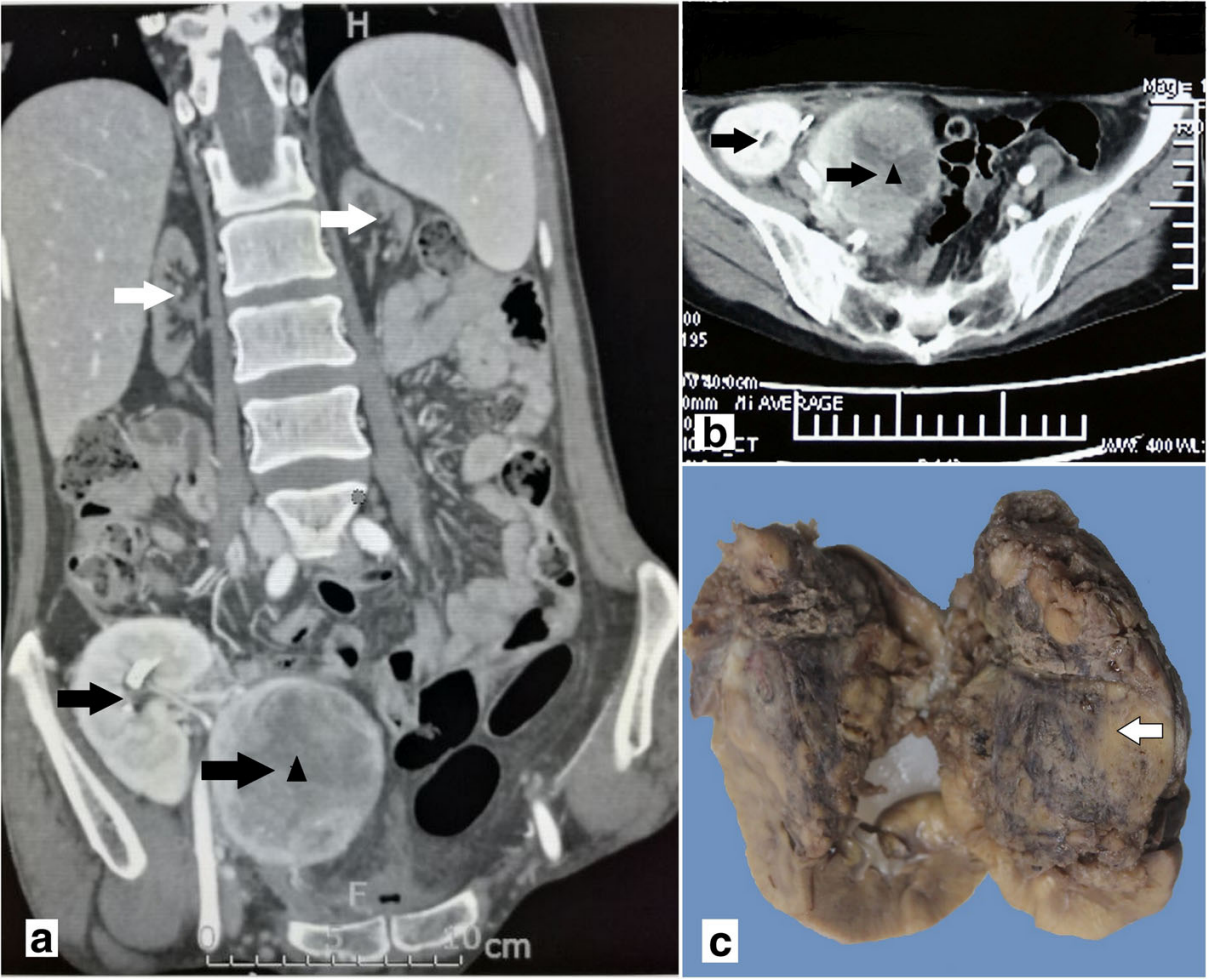
Computed tomography (CT) scan revealed that both original kidneys exhibited atrophy (**a**, white arrows) and a large mass (**a** and **b**, triangle) measuring 7.4 × 6.3 × 6.0 cm with a mixed density in the upper pole of the inner transplanted kidney (**a** and **b**, black arrows). **c**. Macroscopic features of MRT of the transplanted kidney. The cut surface of the mass was white to grayish (arrow)

Dual kidneys were transplanted in the right iliac fossa of the recipient (Fig. 1a and b, black arrows), and the original kidneys were not removed (Fig. 1a, white arrows). Her immediate post-transplant situation was placid. Four months later, she developed hematuria and accelerated graft dysfunction. Ultrasound and computed tomography (CT) showed a 73-mm mass within the enlarged internal transplanted kidney (Fig. 1a and b, triangle), and the external transplanted kidney was unchanged. The enlarged transplanted kidney was, therefore, removed by surgery.

Follow-up revealed that the recipient was alive and well without recurrence 10 months after diagnosis, and the other intact transplanted kidney showed no mass by routine CT scan.

### Pathological findings

The removed transplanted kidney measured 9.0 × 7.9 × 7.5 cm. The cut surface showed a 7.4 × 6.3 × 6.0 cm mass without a capsule located in the upper pole, replacing almost the entire kidney (Fig. 1c). The samples were fixed in 4% formalin and embedded in paraffin. Sections were cut and stained with hematoxylin & eosin (H&E). For immunohistochemistry, the paraffin-embedded tissue blocks were sliced to 3–4 μm thickness. After deparaffinization, antigen retrieval with heat and 3% hydrogen peroxide (H_2_O_2_) methanol solution treatment was done for 30 min to eliminate nonspecific reaction with proteins. The primary antibodies applied are given in Table 1. Immunostaining was performed by an enhancement method based on repetitive microwave heating of slides that were placed into 0.01 M citrate buffer at pH 6.0. Binding of primary antibodies was visualized with an Envision two-step method. Diaminobenzidine was used as chromogen, and nuclei were stained with Mayer’s hematoxylin. Appropriate positive controls were included.

**Table 1:** Antibodies and dilutions used in the evaluation of malignant rhabdoid tumor in transplanted kidney

Antibody	Dilution	Source
Pancytokeratin	1:100	Dako
EMA	1:50	Dako
Vimentin	1:100	Novocastra
INI-1	1:100	Dako
CD99	1:50	Dako
WT-1	1:100	Dako
Desmin	1:200	Dako
Myogenin	1:100	Dako
MyoD1	1:100	Dako
CAIX	1:100	Santa Cruz
CD34	1:100	Dako
CD56	1:100	Novocastra
CgA	1:100	Dako
Syn	1:100	Dako
GATA-3	1:100	Santa Cruz
CD10	1:100	Novocastra
CD117	1:200	Dako
CK5/6	1:200	ZYMED
P63	1:25	Novocastra
AMACR	1:100	Dako
TFE-3	1:200	Santa Cruz
S-100	1:1000	Dako
HMB45	1:100	Dako
MelanA	1:200	Dako
CD38	1:100	Novocastra
CD138	1:100	Dako
KI67	1:30	Novocastra

**Fig. 2:**
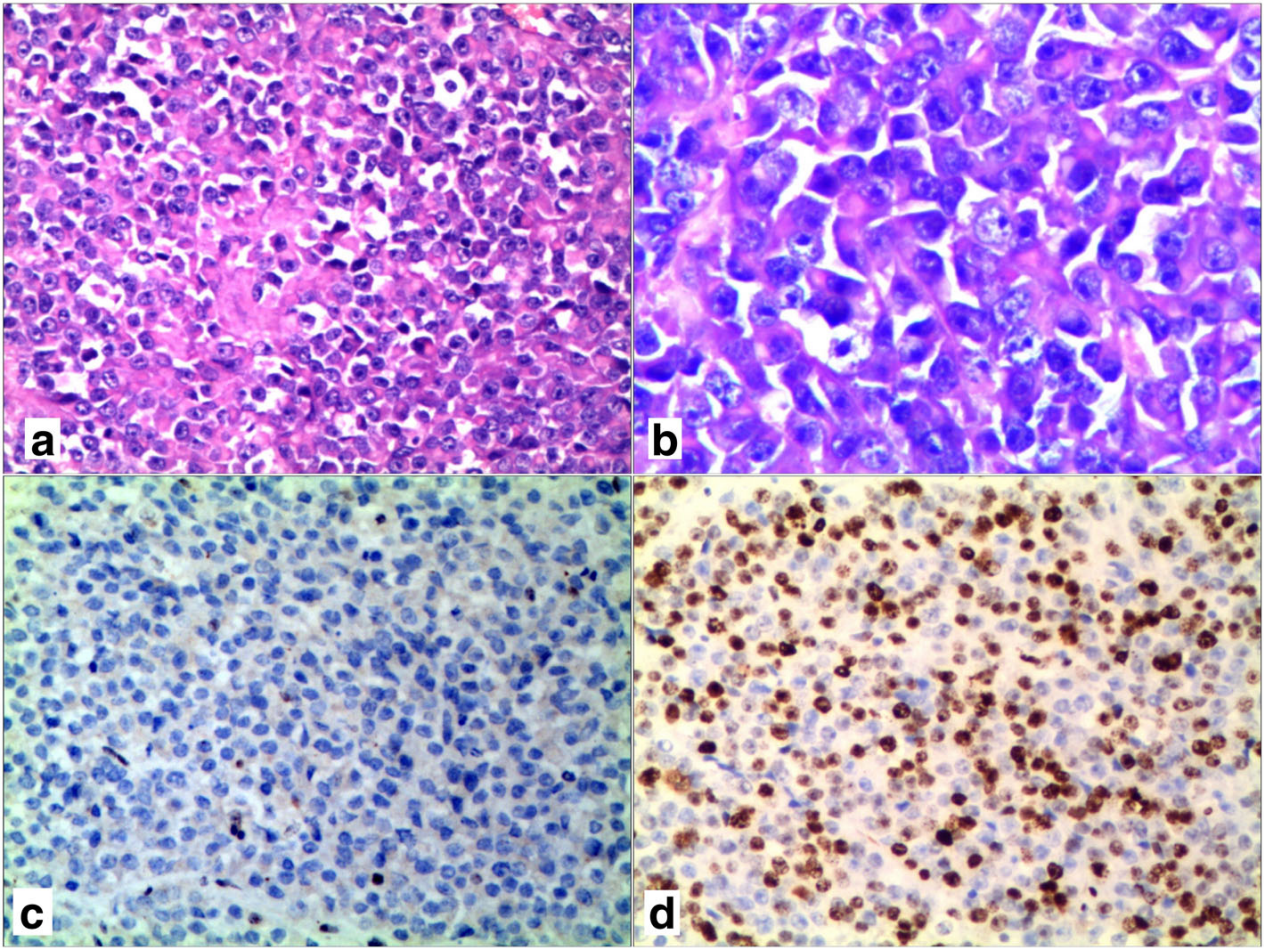
The tumor cells showed patternless sheets or nests of noncohesive, uniform, round and oval tumor cells having eccentric nuclei with macro-nucleoli and abundant eosinophilic cytoplasm (**a**, H&E × 200 and **b**, H&E × 400). **c** tumor cells showing loss of staining for INI1 immunohistochemically. **d** The Ki67 labeling index showed high proliferation rates of the tumor cells immunohistochemically

Microscopic examination showed patternless sheets or nests of noncohesive, uniform, round and oval tumor cells having eccentric nuclei with macro-nucleoli and abundant eosinophilic cytoplasm (Fig. 2a, b). Some of the neoplastic cells had round, eosinophilic, hyaline-whorled paranuclear cytoplasmic inclusions.

### Immunohistochemistry and molecular analysis

Immunohistochemically, the tumor cells were diffusely positive for vimentin and CD99, focally positive for epithelial membrane antigen (EMA) and cytokeratin, but negative for SMARCB1/INI1(Fig. 2c). All other markers (WT-1, desmin, myogenin, myogenic differentiation 1(MyoD1), carbonic anhydrase IX(CAIX), CD34, CD56, chromogranin A (CgA), synaptophysin (Syn), GATA binding protein 3 (GATA-3), CD10, CK5/6, P63, CD117, P504s, transcription factor binding to IGHM enhancer 3 (TFE-3), S-100, HMB45, MelanA, CD38, and CD138) were not expressed in the tumor cells. The Ki67 labeling index was approximately 80% (Fig. 2d).

For FISH analysis of *SMARCB1/INI1* and sex chromosomes, paraffin-embedded 5-μm sections were deparaffinized. A probe specific for *SMARCB1/INI1* (Empire Genomics, NY, USA) and a dual-color interphase FISH probe set for the X centromere (CEP X) and Y centromere (CEP Y) (GP Medical Technologies Inc., Beijing, China) were used to detect any abnormality of *SMARCB1/INI1* and male (XY) donor or female (XX) recipient cells present in the kidney tumor specimen according to the manufacturers’ protocols. The fluorescence signals were analyzed using an Olympus BX51 fluorescence microscope (Olympus, Tokyo, Japan) equipped with appropriate filters and imaged using Vysis software. At least 200 cells were scored.

**Fig. 3:**
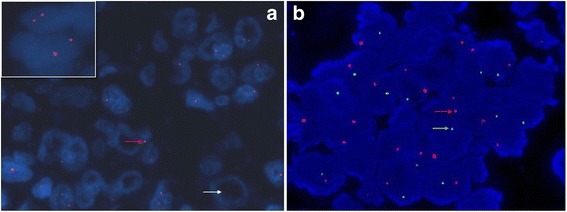
Fluorescence in situ hybridization (FISH) analysis: **a** Deletion of *SMARCB1/INI1* is evidenced by loss of one (red arrow) or both (white arrow) probe signals (red) in tumor nuclei, while two copies are retained in the nuclei of normal renal tubular epithelial cells (insert). **b** FISH analysis of sex chromosomes showed tumor cells had a male gonosomal complement (positive results for the X (green arrow) and Y chromosomes (red arrow), consistent with donor origin)

FISH analysis showed that *SMARCB1/INI1* was deleted (Fig. 3a), and most of the tumor cells had a male gonosomal complement with an X (green) and a Y (red) chromosome, which was consistent with donor origin (Fig. 3b).

For the short tandem repeat (STR)-based concordance study, genomic DNA was extracted from tumor tissue, normal renal tissue samples of the transplanted kidney and skeletal muscle of the recipient using the Chelex-100 protocol and subsequently quantified with the Nanodrop 2000 spectrophotometer (Thermo Fisher Scientific, MA, USA). Multiplex genotyping was performed with the EX22 STR kit (AGCU ScienTech Incorporation, Wuxi, China) and the AmpFLSTR® Identifiler® PCR Amplification kit (Thermo Fisher Scientific, MA, USA) on the case samples according to the manufacturers’ recommendations. The experimental procedures followed internal laboratory control standards and kit controls.

**Fig. 4:**
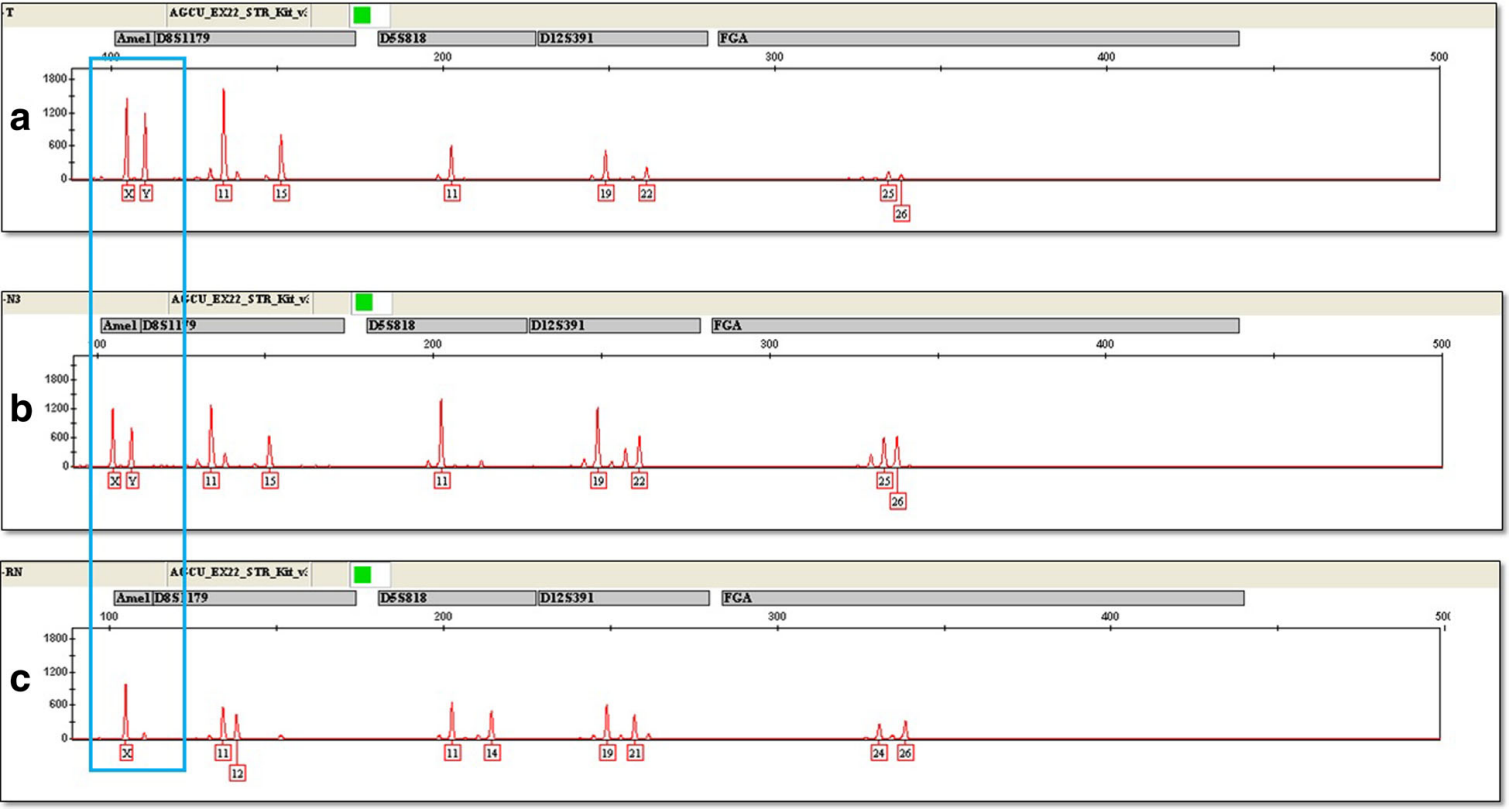
Short tandem repeat (STR)-based concordance study of the 22 microsatellite markers tested, differences were found between tumor (**a**) and skeletal muscle from the recipient (**c**) in all 22 informative alleles, and a Y chromosome (blue box) was detected, indicating that the MRT was derived from the donor cells (**b**)

The STR results showed that of the 22 microsatellite markers tested, differences were found between tumor cells (Fig. 4a) and skeletal muscle from the recipient (Fig. 4c) in all 22 informative alleles, and a Y chromosome was detected in the tumor cells. We concluded that the tumor was derived from the donor (Fig. 4b).

## Discussion

Primary tumors in the kidney allograft recipients have often been reported since the advent of potent immunosuppression. The most common malignant tumors of organ transplant recipients are post-transplant lymphoproliferative diseases and skin cancer [5]. To our knowledge, this is the first case of MRT in a kidney transplanted into an adult.

MRT is a rare neoplasm that occurs mainly in the kidney in children less than 1 year of age, with an aggressive clinical course [6]. Some cases with histologic appearance similar to that arising in the kidney have been described in virtually every extrarenal anatomic site, including soft tissue, retroperitoneum, mediastinum, orbit, gastrointestinal system, uterus, and most prominently the CNS, where they are referred to as atypical teratoid/rhabdoid tumors (AT/RT) [7]. As a distinct and unique type of malignant tumor, MRT has the characteristic appearance of patternless sheets of noncohesive cells with abundant cytoplasm and eosinophilic inclusions, as well as specific molecular aberrations involving *SMARCB1* (hSNF5/INI1), which can be identified by a lack of staining with INI1 immunohistochemically. Fewer than 10 cases of MRTs are reported in adult patients in the English-language literature [8, 9], and only one case of MRT in the native kidney in an adult patient following kidney transplantation was reported [10]. In the present study, the mass in the transplanted kidney was diagnosed as MRT based on the histopathological features and immunohistochemical findings.

Other primary renal neoplasms and certain epithelioid or rhabdoid cell lesions arising from the kidney and urinary tract should be distinguished from MRT upon diagnosis. Nephroblastoma (Wilms tumor), a common solid tumor of childhood, typically shows a distinct triphasic pattern with blastema, stromal, and epithelial components and without *INI1* deficiency. Epithelioid sarcoma is a malignant tumor affecting an older age group than MRT, with prominent rhabdoid cells and loss of nuclear INI1 in most cases, and it often shows CD34-positive staining, mainly located in the extremities. Other malignancies, including rhabdomyosarcoma, myoepithelioma in soft tissue, epithelioid angiosarcoma, and epithelioid malignant peripheral nerve sheath tumor (MPNST), can be distinguished from MRT by their specific markers and INI1 expression. Loss of INI1 is also helpful to differentiate MRT from other primary renal neoplasms with rhabdoid differentiation, including clear cell renal carcinoma, chromophobe cell renal carcinoma, and other malignances in the urinary system [11, 12].

Due to the rarity of MRT in transplanted kidney, FISH and STR analysis were used to determine the tumor origin in this case. The results showed a Y chromosome in the tumor cells, which was consistent with donor origin. In general, malignant tumors in allografts can develop in two ways: de novo development in the allograft of an immunosuppressed recipient [1] and donor-to-recipient malignancy transmission [13, 14]. In the present case, the recipient’s medical record revealed that the deceased donor was a 56-day-old infant who died of a CNS tumor (suspected astrocytoma without pathology or autopsy evidence), and there was no evidence of a mass in the allograft by imaging examination before transplantation.

There was no history of rhabdoid tumors in the donor family history.

Although we cannot refute the diagnosis of astrocytoma and prove the presence of AT/RT, we strongly suspect the CNS tumor of the infant donor was an AT/RT, a tumor that predominantly affects infants and young children and has histological features similar to MRT in kidney, with poor prognosis. FISH and STR analysis of the recipient’s tumor was consistent with donor origin. Therefore, we consider donor-to-recipient malignancy transmission likely in this case, making this the first report of MRT in a renal allograft of an adult recipient following renal transplantation.

Donor-to-recipient malignancy transmission could occur in two ways. First, there could be some metastatic cells in the transplanted kidney that come from a primary tumor in another organ and then transfer in the organ recipient. Second, post-transplant lymphoproliferative disorder, the most common post-transplant malignancy, could come from malignant change of passenger leukocytes in the transplanted organ. We favor the former explanation in this case, as it was likely that the tumor cells from AT/RT in the CNS of the pediatric donor metastasized to the kidney, which was transplanted to the adult female recipient.

According to strategy and rules for organ screening and acknowledgment before transplantation, donors with a history of lung cancers, sarcomas and grade IV CNS neoplasms are considered unacceptable, but donors with a past with grade I-II CNS tumors are acceptable. In the present case, the donor, a 56-day-old infant who died of a CNS tumor with a suspected diagnosis of astrocytoma, was not reasonable because AT/RT should have been considered in the differential diagnosis before the transplantation.

Since the number of patients requiring transplantation therapy is growing, there is increasing demand for donor organs. The expansion of the general donor pool has led to the inclusion of donors of extreme ages and donors who may possibly transmit disorder to the recipients. In any case, the peripheral donors’ organ utility is frequently connected with a more serious danger of undiagnosed disease including malignant tumors and some infections [15]. Thus, the evaluation of infants or very young children with CNS tumors as donor candidates for kidney transplantation should be investigated for the synchronous presence of kidney tumors.

## Conclusion

We report a unique case of MRT in an adult renal allograft recipient after kidney transplantation with characteristic histologic features and loss of INI1 expression. This report highlights the importance of the criteria for selection of donors to screen possible malignant tumors transmission in living and dead donor transplants.

## Abbreviations

AT/RT: Atypical teratoid/rhabdoid tumor
CAIX: Carbonic anhydrase IX
CEP X: X centromere
CEP Y: Y centromere
CgA: Chromogranin A
CNS: Central nervous system
CT: Computed tomography
EMA: Epithelial membrane antigen
FISH: Fluorescence in situ hybridization
GATA-3: GATA binding protein 3
H&E: Hematoxylin & eosin
INI1: Integrase interactor 1
MPNST: Malignant peripheral nerve sheath tumor
MRT: Malignant rhabdoid tumor
MyoD1: Myogenic differentiation 1
SMARCB1: SWI/SNF-related matrix-associated actin-dependent regulator of chromatin subfamily B member 1
STR: Short tandem repeat
Syn: Synaptophysin
TFE3: Transcription factor binding to IGHM enhancer 3
